# Construction of a Normalized Library and Screening of Transcriptional Regulators of the *cas5* Gene in *Corynespora cassiicola*

**DOI:** 10.3390/microorganisms14010129

**Published:** 2026-01-07

**Authors:** Baoping Zhu, Guohao Hu, Ziping Yang, Raja Asad Ali Khan, Musharaf Ahmad, Muhammad Zaryab Khalid, Tong Liu, Jumei Hou

**Affiliations:** 1 Sanya Institute of Breeding and Multiplication, Engineering Center of Agricultural Microbial Preparation Research and Development of Hainan, School of Tropical Agriculture and Forestry, Hainan University, Haikou 570228, China; zhubaoping1228@163.com (B.Z.); 18389867742@163.com (G.H.); asadraja@aup.edu.pk (R.A.A.K.); zaryabkhalid0003@hotmail.com (M.Z.K.); 2 Zhanjiang Key Laboratory of Tropical Crop Genetic Improvement, South Subtropical Crops Research Institute, Chinese Academy of Tropical Agricultural Sciences, Zhanjiang 524091, China; yangziping302@163.com; 3 Department of Plant Pathology, The University of Agriculture, Peshawar 25130, Pakistan; makpk64@aup.edu.pk

**Keywords:** rubber tree defoliation, bZIP gene, Yeast one-hybrid

## Abstract

In tropical rubber-growing regions, *Corynespora* leaf fall disease stands as a predominant and economically significant threat to rubber trees. The toxin protein encoded by the *cas5* gene is the main pathogenic factor of *Corynespora cassiicola*. To identify transcription factors capable of binding with the *cas5* gene promoter sequence of *C. cassiicola*, the promoter of the *cas5* gene was predicted by bioinformatics, and the 1000 bp promoter of the *cas5* gene was isolated to construct a yeast one-hybrid bait vector for self-activation detection. A yeast one-hybrid cDNA expression library of *C. cassiicola* was constructed to screen for potential transcriptional regulators interacting with the 1000 bp promoter of the *cas5* gene. The transcriptional regulators interacting with the *cas5* gene were determined by the yeast one-hybrid (Y1H) point-to-point verification experiment. Y1H results showed that the bait vector did not have self-activation. The cDNA library had a titer of 2.516 × 10^8^ cfu/mL and a total clone count of 5.032 × 10^8^. Screening identified 30 candidate transcriptional regulators. Through point-to-point yeast one-hybrid verification, only one of the 30 candidate transcription factors (named CcbZIP3629) showed interaction with *cas5.* Molecular docking was performed using the AlphaFold3-predicted structure of CcbZIP3629, which revealed its binding to two ACGT core motifs within the promoter. These findings provide the groundwork for elucidating the regulatory mechanism of the *cas5* gene, particularly by which CcbZIP3629 mediates the expression of the Cc-Cas5 toxin.

## 1. Introduction

*Corynespora* leaf fall disease, caused by *Corynespora cassiicola*, is one of the most destructive diseases in rubber-producing areas worldwide. It severely compromises rubber tree development and latex production in tropical regions and poses a significant challenge to the sustainability of natural rubber production [1]. This disease is prevalent in rubber-growing countries across Southeast Asia, West Africa, and Latin America. Infection can lead to extensive leaf fall, annual defoliation, and even death of young trees. These effects seriously affect the photosynthesis and growth, resulting in a significant yield reduction [2,3,4,5]. At present, the current research on the pathogen’s mechanism focuses mainly on the identification, expression, and detection of toxin proteins, with limited studies in other aspects.

The pathogenicity of *C. cassiicola* is closely related to the diversity of its secreted cassiicolin toxin proteins [6]. The Cassiicolin toxins, encoded by the *cas* genes, are O-glycosylated proteins composed of 27 amino acid residues, which can induce cell damage in rubber tree leaves identifical to that caused by the fungus itself and exhibit the same host selectivity [7,8,9,10]. At present, seven Cassiicolin subtypes (*cas0* to *cas6*) have been identified. According to the specific *cas* genes carried and expressed by different isolates, it is hypothesized that these specific Cas genes could underlie the pathogen’s host specificity [11,12]. The function of *C. cassiicola* pathogenic genes is host-specific. For instance, the deletion of the *cas2* gene has no effect on cucumber or strawberry strains, while deletion of the *cas1* gene leads to a decreased or complete loss of pathogenicity in rubber tree strains [13]. The identification of *C. cassiicola* in five cities and counties of Hainan showed that the *cas5* gene was detected in 35 isolated strains, and no other types of toxin genes were found. Strains and toxin type analysis revealed that isolates containing the *cas5* subtype originated from rubber trees and represent the dominant population on rubber trees in Hainan [14].

Transcription factors control gene transcription through specific attachment to promoter DNA sequences. In fungi, universal transcription factors (GTFs) bind to promoters to form an initiation complex and recruit RNA polymerase II, relying on conserved elements for precise localization. Promoter function studies are often combined with bioinformatics prediction and experimental verification (such as the effector protein PsCRN108 targeting the promoter to enhance pathogenicity) [15,16,17,18]. These regulatory molecules modulate transcriptional output through their association with cis-regulatory elements in the non-coding portions of their target genes, thereby controlling transcriptional timing and intensity [19,20,21]. However, the specific transcription factors and regulatory networks controlling toxin protein expression in *C. cassiicola* have not been reported.

Therefore, in order to elucidate the transcription factors of the *cas5* gene in *C. cassiicola*, this study analyzed and isolated its promoter. We constructed a yeast one-hybrid bait vector and cDNA expression library to identify proteins that interact with the promoter sequence of the *cas5* gene. SMART^®^ technology and the DSN homogenization method were used to construct a high-quality cDNA library to increase the proportion of full-length clones and balance transcripts with different abundances. The findings of this study lay the groundwork for elucidating the transcriptional regulation of the *cas5* gene, and lay a foundation for further exploring the transcriptional regulation mechanism of cas5 toxin protein biosynthesis in *C. cassiicola*.

## 2. Materials and Methods

### 2.1. Preservation and Culture of Strains

The pathogen of *C. cassiicola* on a rubber tree stored at 4 °C was inoculated onto Potato Dextrose Agar (PDA) plates and cultured at 28 °C for 4 d. The mycelium on Potato Dextrose Agar (PDA) plates was scraped and placed in a mortar, frozen in liquid nitrogen, and ground to extract RNA.

### 2.2. Bioinformatics Prediction of cas5 Promoter

The analysis of the cis-regulatory elements in the upstream regulatory region of the *cas5* gene was performed utilizing the PlantCare software (https://bioinformatics.psb.ugent.be/webtools/plantcare/html/, accessed on 30 May 2025) [22]. Although some tools (such as PlantCARE) are mainly developed for plants, the core cis-acting elements identified are relatively conserved in eukaryotes and have been successfully applied to a number of fungal studies [23]. This study combines a variety of tools to reduce species bias. Potential transcription factor binding motifs within the promoter were identified via PlantPan 4.0 (https://plantpan.itps.ncku.edu.tw/plantpan4/promoter_results.php, accessed on 30 May 2025). The MethPrimer tool (http://www.urogene.org/methprimer/, accessed on 30 May 2025) was used to predict CpG islands [24]. The physiochemical properties of the transcription factor CcbZIP3629 protein were predicted using the Expasy ProParam tool (https://web.expasy.org/protparam/, accessed on 30 May 2025). SignalP 5.0 (https://services.healthtech.dtu.dk/services/SignalP-5.0/, accessed on 30 May 2025) was employed to detect the presence of a signal peptide. TMHMM 2.0 (https://services.healthtech.dtu.dk/services/TMHMM-2.0/, accessed on 30 May 2025) was employed to predict the transmembrane structure of the protein. The SMART database (http://smart.embl-heidelberg.de/, accessed on 30 May 2025) was employed to predict the transcription factor family and its DNA-binding characteristics. The NetPhos 3.1 Server (http://www.cbs.dtu.dk/services/NetPhos/, accessed on 30 May 2025) was used to analyze the phosphorylation sites within the protein. The SOPMA tool (http://npsa-pbil.ibcp.fr/cgi-bin/npsa_automat.pl?page=npsa_sopma.html, accessed on 30 May 2025) was applied to determine the secondary structure of the protein. The tertiary structure of the CcbZIP3629 protein was predicted using the SWISS-MODEL tool (https://swissmodel.expasy.org/interactive/, accessed on 30 May 2025). Furthermore, the subcellular localization of the transcription factor was determined using the Wolf PSORT website (https://www.genscript.com/wolf-psort.html accessed on 30 May 2025). Molecular docking was performed using AlphaFold3 (https://alphafoldserver.com/, accessed on 30 May 2025), and visualization was completed by PyMOL 3.1.

### 2.3. RNA Extraction and Yeast One-Hybrid (Y1H)-Normalized Library Construction

In this study, SMART^®^ technology was used to construct a cDNA library. This technology can effectively enrich cDNA with a complete 5′ end by template conversion, thereby significantly improving the efficiency of full-length gene cloning. RNAprep Pure Micro Kit (TianGen, Beijing, China) was used to extract the total RNA of *C*. *cassiicola*. The library construction method references the SMART cDNA Library Construction Kit (Takara Bio, Mountain View, CA, USA). CDS III/3′ PCR Primer and SMART IV Oligonucleotide (CDS III/3′ PCR Primer, SMART IV Oligonucleotide, Table 1) were used for reverse transcription to synthesize cDNA. Gene-specific primers P1/P2/P3-F and P4-R (P1/P2/P3-F, P4-R, Table 1) were used for cDNA amplification. In this study, primers were designed using the molecular biology software SnapGene (version 4.2.1). With the exception of the universal primers T7-F and AD-R, all other primers were designed through this software. For first-strand cDNA synthesis, 2 µL of mRNA was mixed with 1 µL of CDS III/3′ PCR Primer, 1 µL of SMART IV Oligonucleotide, and 1 µL of DEPC-H_2_O. The reaction underwent a 3 min incubation at 72 °C, followed by immediate transfer to an ice bath. Subsequently, the mixture was supplemented with 1 µL of DTT (20 mM), 2 µL of 5× First-Strand Buffer, 1 µL of dNTP Mix (10 mM), and 1 µL of MMLV reverse transcriptase. The reaction was then incubated at 42 °C for 90 min, followed by 70 °C for 15 min, and then cooled to room temperature. Later, 1 µL of RNase H was added and the mixture was incubated at 37 °C for 30 min. For PCR amplification, a reaction mixture was prepared containing 2 µL of cDNA, 25 µL of 2× PCR Mix, 2 µL of P4-R, 2 µL of P1/P2/P3-F, and 19 µL of ddH_2_O in a 0.2 mL PCR tube. The amplification protocol was as follows: 95 °C for 3 min; 30 cycles of 95 °C for 15 s, 60 °C for 15 s, and 72 °C for 6 min; followed by a final extension at 72 °C for 10 min. In order to reduce the impact of high-abundance transcripts, the purified double-stranded cDNA was processed using a homogenization method based on Kamchatka crab duplex-specific nuclease (DSN), specifically referring to the scheme of Zhulidov et al. [25]. A 50 μL amplification system was established using 1 μL of DSN digestion product as a template, including 40.5 μL of ddH_2_O, 5 μL of 10 × Advantage 2 PCR buffer, 1 μL of 50 × dNTP Mix, 1.5 μL of primer M1, and 1 μL of 50 × Advantage 2 polymerase Mix. The reaction procedure is as follows: pre-denaturation at 95 °C for 1 min; subsequent cycle reaction: 95 °C for 15 s, 66 °C for 20 s, and 72 °C for 3 min, for a total appropriate number of cycles; final extension at 72 °C for 5 min. The PCR products were purified using DNA Clean Beads and small fragments were removed using Clontech CHROMA SPIN TM + TE-1000 Columns (Takara, San Jose, CA, USA). Smart technology was used for the construction of the Y1H library. The purified cDNA was homologously recombined with the linearized pGADT7 plasmid, and then the plasmid was transformed into *E. coli*. To identify the recombinant clones, 24 single colonies were randomly selected and resuspended in sterile water as PCR templates. The colony PCR reaction system (20 μL) was as follows: 8 μL of nuclease-free water (ddH_2_O), 10 μL of 2 × PCR premix Mix (2 × PCR Mix), 1 μL of T7 forward primer (T7-F, 10 μM), and 1 μL of 3′-anchored primer (3′AD, 10 μM). The PCR reaction was carried out in a thermal cycler, and the program was set as follows: pre-denaturation at 95 °C for 3 min; subsequently, 35 cycles were carried out: 95 °C denaturation for 15 s, 55 °C annealing for 15 s, and 72 °C extension for 1 min; and a 72 °C final extension of 5 min after the end of the cycle. After the reaction was completed, 10 μL of PCR products were taken and detected by 1% agarose gel electrophoresis to preliminarily screen positive clones according to the size of the amplified fragments. After the YIH library was successfully constructed, the quality of the library was identified by measuring the titer and average insertion length of the library. The quality of the library is related to the library capacity and the length of the library insertion fragment. The library capacity is greater than 1 × 10^6^ cfu to ensure that positive clones are obtained. When measuring the titer of the library, 10 μL of the bacterial culture medium was diluted 10,000 times, and 100 μL was coated on the LB plate containing ampicillin and cultured overnight at 37 °C. After the transformation products were plate-cultured, the colonies on all plates were counted by the manual counting method, and the titer of the library was calculated.

Library titer (cfu/mL) = number of clones/0.1 mL × 10,000

Library capacity (cfu) = titer × bacterial volume

### 2.4. Self-Activation Identification of Bait Vector

The pHIS2-Cas5-1000 plasmid, constructed in previous studies, served as the bait to probe the *C. cassiicola* cDNA library in yeast one-hybrid analysis. After detecting multiple reporter genes, the DNA of positive clones was sequenced and analyzed through BLAST alignment, which identified proteins potentially interacting with pHIS2-Cas5-1000 (https://blast.ncbi.nlm.nih.gov/Blast.cgi, accessed on 20 May 2024). 3-AT, a competitive inhibitor of yeast HIS3 protein, was employed to inhibit the leakage expression of the HIS3 gene. During the activation process, the addition of a maximum 50 mM 3-AT in the medium effectively inhibited self-activation and reduced false positives in library screening. For the assays, the pHIS2-Cas5-1000 plasmid and pGADT7 plasmid, as well as positive control plasmids pHIS2-p53 and pGAD53m, were transformed into Y187 yeast strains. The transformed strains were spread on SD/-Trp and SD/-Trp/-Leu plates, respectively. Three individual colonies were randomly selected from each transformation plate, diluted, and spread onto the SD/-Trp/-Leu/-His+media supplemented with 3-AT (add 0, 2.5, 5, 10, 20, 30, 40, 50, 75, and 100 mM). The plates were then subjected to incubation at 30 °C for three days. There were three replicates across the experiments.

### 2.5. Y1H-Based Identification of Candidate Transcriptional Regulators of cas5 Gene

The library plasmid was transformed into Y187 yeast competent cells containing the correct pHIS2-1000 bait plasmid by the lithium acetate method. The brief steps are as follows: first, the yeast strain was pre-cultured in SD/-Trp medium, and then transferred to YPDA medium to culture to OD_600_ = 0.6. The bacteria were collected by centrifugation and washed with sterile water and 0.1 M LiAc solution in turn. Then, 25 μg of library plasmid DNA was mixed with 9.6 mL of 50%PEG3350, 1.44 mL of 1 M LiAc, and 300 μL of denatured salmon sperm single-stranded DNA (10 mg/mL), and fully mixed with competent cells. Subsequently, the cells were incubated at 30 °C for 30 min, heat shocked at 42 °C for 25 min, and resuscitated at 30 °C for 1 h. After transformation, the cells were resuspended with sterile water. A small amount was coated on the SD-TL plate to calculate the conversion efficiency, and the remaining bacterial solution was uniformly coated on 20 SD/-Trp/-Leu/-His (containing X mM 3AT) plates. Cultured at 30 °C for 3–7 days, colony growth was observed. The *C. cassiicola* cDNA library plasmid and pHIS2-Cas5-1000 bait plasmid were co-transformed into Y187 yeast and coated on the SD/-Trp/-Leu/-His+X mM 3AT plate. The positive transformants grown on the screening plate were collected and sequenced by NGS. Then, the sequencing results were analyzed by NCBI blast X to obtain the candidate transcriptional regulators of the *cas5* gene. The whole-genome sequence data reported in this paper have been deposited in the Genome Warehouse in the National Genomics Data Center [26,27], Beijing Institute of Genomics, Chinese Academy of Sciences/China National Center for Bioinformation, under an accession number that is publicly accessible at https://ngdc.cncb.ac.cn/gwh (accessed on 30 May 2025).

### 2.6. Point-to-Point Verification of the Interaction Between Candidate Transcription Factors and the cas5 Gene Promoter

The candidate transcriptional regulatory molecules obtained above were inserted into the yeast expression vector pGADT7 and transformed into DH5α for sequencing. Correct colonies were selected, and plasmids were extracted. The following plasmid combinations were co-transformed into Y187 yeast competent cells: the pGADT7 empty plasmid with the pHIS2-Cas5-1000 plasmid, pHIS2-p53 with pGAD53m (positive control), and the pGADT7-candidate transcription factor with pHIS2-Cas5-1000. Transformants were plated on SD/-Trp/-Leu medium, and correct colonies were verified by PCR. Verified colonies were then cultured in YPDA medium for 18–24 h. The OD_600_ of the colonies was then adjusted to 0.2 and plated on SD/-Trp/-Leu/-His medium supplemented with X mM 3-AT to access the transcription factors interacting with the *cas5* gene. The interaction between the transcription factor and *cas5* promoter was verified by at least three independent yeast transformation experiments (biological replicates), and three technical replicates were set up in each experiment.

## 3. Results

### 3.1. Bioinformatics Prediction of cas5 Gene Promoter Sequence

**Figure 1:**
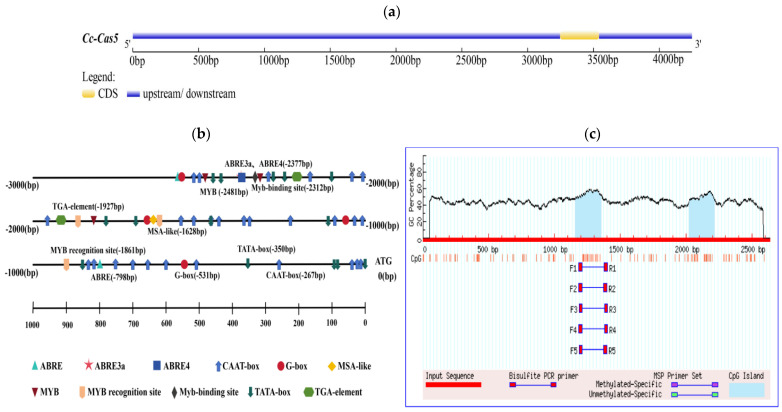
Identification of *cas5* gene promoter in *C. cassiicola* of rubber tree. (**a**) Upstream and downstream of *cas5* gene. (**b**) The location of various cis-regulatory elements of the *cas5* gene promoter, such as TATA-BOX and CAAT-BOX, was analyzed in detail. (**c**) Prediction of CpG islands in the *cas5* promoter. The graph identifies two CpG islands within the promoter sequence.

The *cas5* gene promoter sequence was analyzed using the PlantCARE online database, which predicted a variety of cis-regulatoryregulatory elements. The results showed the detection of multiple TATA-boxes and CAAT-boxes, indicating that the promoter possesses a typical core promoter structure and high transcriptional activity, which is a prerequisite for basal transcription (Figure 1a,b). Further analysis using the PlantPan database revealed that the promoter contains a total of 2156 transcription factor binding sites. These sites belong to diverse transcription factor families, with the major groups including MYB, bZIP, WRKY, NAC/NAM, bHLH, HD-ZIP, AP2/ERF, C2H2, GATA, and Dof. Additionally, sites were associated with other transcription factors that are not clearly classified or have multiple functions. Concurrently, MethPrimer was utilized to predict the presence of two CpG islands in the *cas* gene promoter, located at 1158–1356 bp and 2021–2213 bp (Figure 1c). Based on these findings, a 1000 bp promoter fragment was selected for subsequent experimentation.

### 3.2. Construction of Y1H Library and Identification of Bait Vector Self-Activation

**Figure 2:**
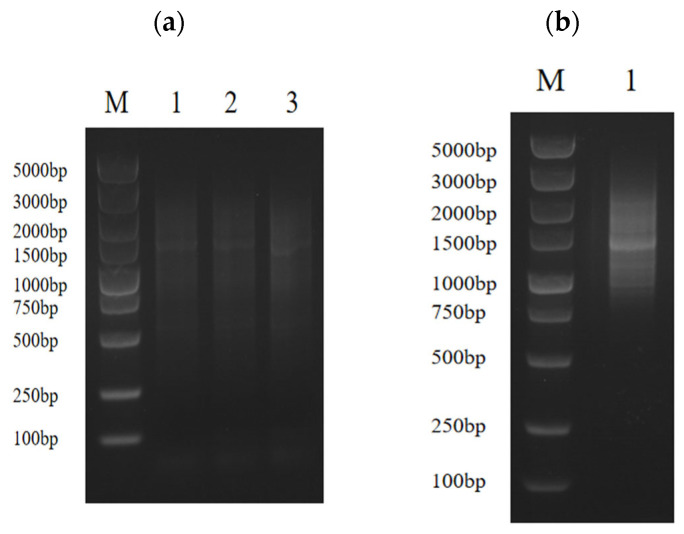
(**a**) The quality of ds cDNA was detected by agarose gel electrophoresis, M: marker; 1: P1-F/P4-R amplification of ds cDNA; 2: P2-F/P4-R amplification of ds cDNA; 3: P3-F/P4-R amplification of ds cDNA. (**b**) ds cDNA purification, M: marker; 1: three ds cDNAs were mixed and homogenized and small fragments were removed.

In order to determine whether the obtained ds cDNA was synthesized, 5 μL was taken for agarose electrophoresis after PCR. The results showed that the ds cDNA showed a diffuse band, indicating that the quality of the synthesized cDNA was good, and the bands of each size were present (Figure 2a). By taking 5 μL from the purified solution for agarose electrophoresis, homogenization and small-fragment removal were performed. The results showed that the ds cDNA showed a diffuse band and no obvious bright band, indicating that the homogenization was successful. Almost no bands were seen below 500 bp, indicating that the small fragments were removed cleanly (Figure 2b).

**Figure 3:**
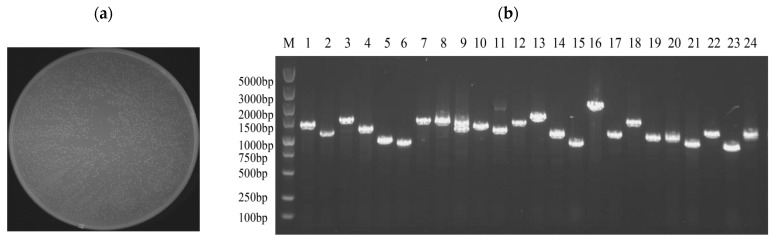
Quality identification of yeast one-hybrid library of *C. cassiicola* in rubber tree. (**a**) The plate containing the 10,000-fold diluted *E. coli* sample showed approximately 2516 colonies upon manual enumeration. (**b**) *E. coli* colony identification PCR, M: marker, 1–24: Randomly selected clones.

The yeast one-hybrid (Y1H) library was successfully constructed using cutting-edge SMART technology, ensuring high-quality and diverse cDNA representation. The number of clones in a single plate section was approximately 629, and the total number of clones on the plate was approximately 2516. Utilizing the established formula, library capacity (cfu/mL) was calculated as the number of clones divided by the coating volume multiplied by the dilution factor. The total number of clones was then determined by multiplying the library capacity by the total volume of the bacterial solution. Based on this calculation, the library capacity was determined to be 2.516 × 10^8^, corresponding to a total of 5.032 × 10^8^ clones (Figure 3a).

The quality of the constructed library was verified by randomly selecting 24 clones from the plate for colony PCR. Following the completion of the PCR, 5 μL was taken for agarose gel electrophoresis. As in Figure 3b, the DNA fragments were approximately 1000 bp in length, confirming an average insert size of approximately 1000 bp for the library fragments and a recombination rate of 100%. These results confirmed that the library met the requirements for the subsequent experiments.

**Figure 4:**
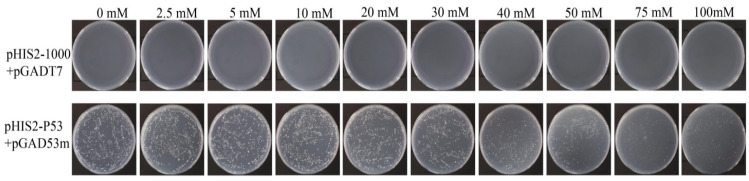
Identification of self-activation of Y1H bait vector. pHIS2-Cas5-1000 plasmid and pGADT7 plasmid were bait vectors, and pHIS2-p53 and pGAD53m were positive control vectors.

In the positive control, the normal growth of the transformants was theoretically unaffected by the 3-AT inhibitor, and the number of colonies was the same as that without 3-AT. However, the observed colony number was about 10% lower than that of 3-AT control, and the growth rate decreased with increasing 3-AT concentration (Figure 4). As shown in Figure 4, the positive control (p53-pGADT7+pGADT7) can grow normally on 0 mM 3-AT medium, indicating that the system works normally. The experimental group (pHIS2-1000+pGADT7) did not grow, which proved that there was no self-activation phenomenon, so 0 mM 3AT was selected for subsequent screening library concentration.

### 3.3. Identification of Candidate Transcription Factors of cas5 by Y1H

**Figure 5:**
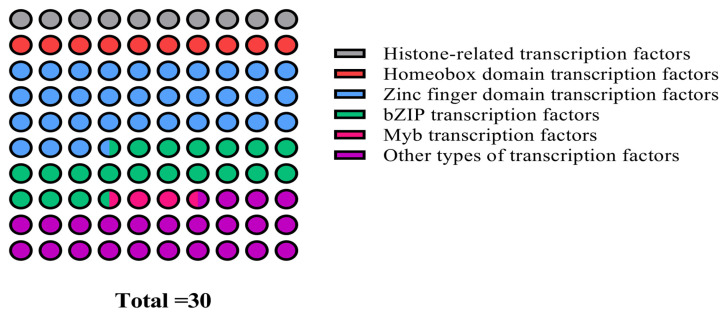
Classification statistics of thirty candidate transcription factors screened by yeast one-hybrid analysis.

Although Y1H is a well-established method for verifying the interaction between protein and DNA, its false positive and sequence repeatability are still high. To address these limitations, a 1000 bp core promoter fragment of the *cas5* gene was strategically selected to construct the bait vector, which effectively reduced self-activation and enhanced the reliability of the yeast one-hybrid library screening. Positive transformants that grew on the SD/-Trp/-Leu/-His+0 mM 3-AT screening plates were collected and subjected to NGS screening. The sequencing results were analyzed by NCBI blast X. Bioinformatics analysis identified a total of 30 candidate potential transcriptional regulators, which were subsequently classified into nine distinct categories (Figure 5). These categories included histone-related transcription factors, homeobox domain transcription factors, zinc finger domain transcription factors, bZIP transcription factors, and other domain-specific transcription factors.

### 3.4. Verification of the Interaction Between cas5 Gene Promoter and Candidate Transcriptional Regulators

**Figure 6:**
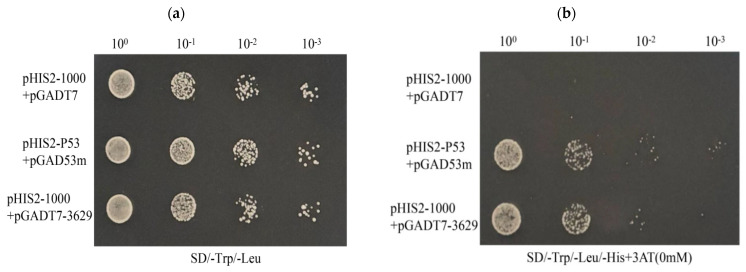
Yeast one-hybrid analysis of the *cas5* gene. The pGADT7 empty plasmid and pHIS2-Cas5-1000 plasmid were used as negative controls, the pHIS2-p53 and pGAD53 m plasmids were used as positive controls, and the pGADT7-3629 plasmid and pHIS2-Cas5-1000 plasmid were used as test groups. (**a**) The growth of the experimental group and the control group on the SD/-Trp/-Leu plate. (**b**) The growth of the experimental group and the control group on the SD/-Trp/-Leu-His medium plate.

Through point-to-point experiments on each candidate transcription factor, we found that one candidate was able to grow on the SD/-Trp/-Leu/-His plate. The growth of the negative control, positive control, and experimental group was observed, demonstrating the reliability of the Y1H transformation system (Figure 6a). Subsequently, the correct colonies were verified by PCR on the SD/-Trp/-Leu/-His plates. The transcription factor that interacts with the *cas5* gene was determined to be CcbZIP3629 (Figure 6b).

### 3.5. Bioinformatics Analysis of the CcbZIP3629 Transcription Factor

**Figure 7:**
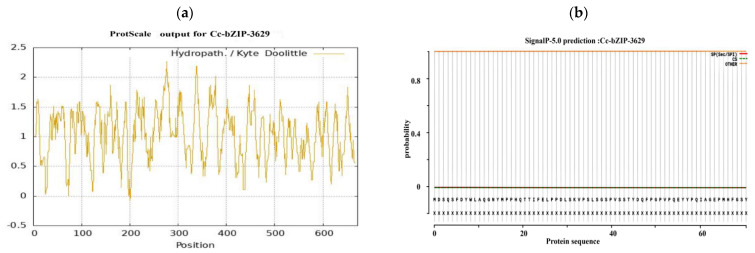

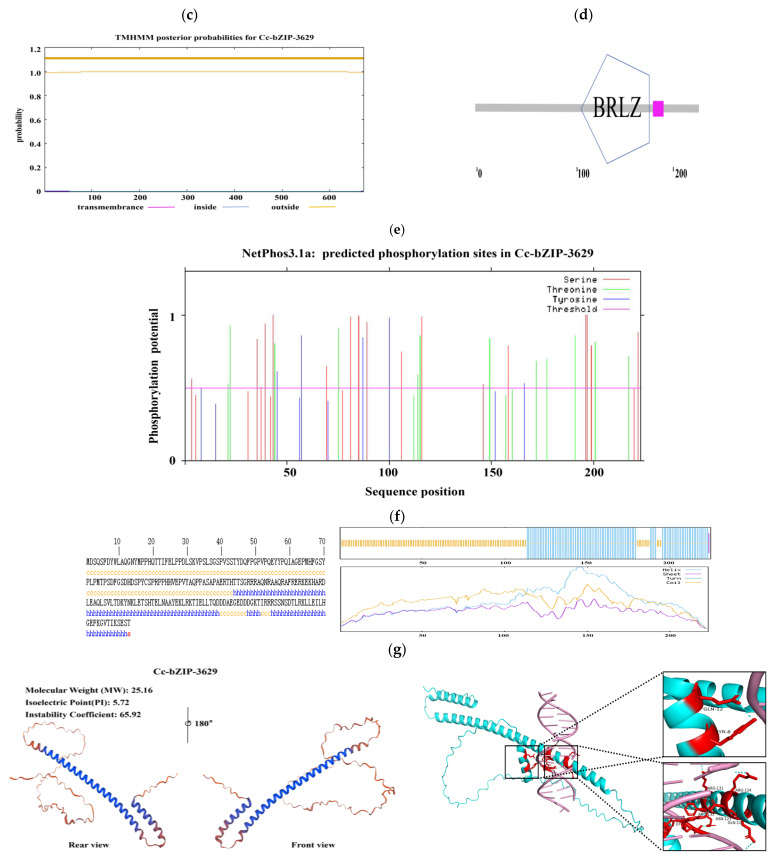
Bioinformatics analysis of the bZIP transcription factors. (**a**) Hydrophilicity prediction. (**b**) Signal peptide prediction. (**c**) Transmembrane structure prediction. (**d**) Transcription factor category prediction. (**e**) Phosphorylation site prediction. (**f**) Protein secondary structure prediction. (**g**) Protein tertiary structure prediction and molecular docking.

Bioinformatics analysis of the CcbZIP3629 transcription factor showed that the full length of the protein was 672 bp, encoding 223 amino acids. The predicted molecular weight was 25.16 kDa and the theoretical isoelectric point (pI) was 5.72 (Figure 7a). Further evaluation of its stability and hydrophilicity showed that the instability coefficient of the protein was 65.92, suggesting that it may be an unstable protein. The aliphatic coefficient was 39.16, and the average hydrophilicity coefficient was −0.965, which showed hydrophilic characteristics. Among them, the 134th amino acid is the most hydrophilic, while the 144th amino acid is the most hydrophobic (Figure 7a). These physicochemical properties provide basic clues for its possible function and intracellular behavior.

In order to explore its subcellular localization and membrane association characteristics, we analyzed the signal peptide and transmembrane domain. The predicted results showed that CcbZIP3629 did not contain a typical signal peptide and transmembrane domain (Figure 7b,c), indicating that it was not a type I or type II transmembrane protein or a protein secreted by the classical secretory pathway. This result is consistent with its subsequent prediction of nuclear localization, but the inference of ‘secreted protein’ needs to be further verified by combining non-classical secretory pathways or other localization signals. The domain identification by SMART database confirmed that CcbZIP3629 contains a typical basic region-leucine zipper (bZIP) domain (Figure 7d), which belongs to the bZIP transcription factor family and may regulate transcription by specifically binding to DNA. This domain usually mediates dimerization and DNA binding, suggesting that it may be involved in gene expression regulation as an activator or suppressor. In addition, a total of 34 potential phosphorylation sites were predicted, including 17 serine, 12 threonine, and 5 tyrosine sites (Figure 7e), suggesting that its function may be dynamically regulated by post-translational modification. The secondary structure prediction showed that α-helix accounted for 43.95% and random coil accounted for 55.61%. The higher proportion of random coil may be related to its structural flexibility and functional regulation. The tertiary structure simulation further revealed that its overall folding was highly similar to the typical bZIP protein structure, and both had conserved α-helix and leucine zipper motifs (Figure 7f), which structurally supported its belonging to the bZIP transcription factor family. Subcellular localization prediction showed that CcbZIP3629 was mainly localized in the nucleus and also distributed in the cytoplasm.

This dual localization suggests that it may have a nuclear-cytoplasmic shuttling ability and may be involved in intracellular signal transduction or regulation processes. Nuclear localization further supports its direct role in gene transcription regulation. Finally, we performed molecular docking simulation of CcbZIP3629 and the ACGT core sequence in the *cas5* gene promoter region (1000 bp) by AlphaFold3. The results showed that the two ACGT core sequences in the promoter may specifically interact with CcbZIP3629 (Figure 7g), which provides a structural basis for CcbZIP3629 to regulate downstream genes by binding to ACGT cis-elements.

## 4. Discussion

*Corynespora* leaf fall disease is the most common and serious disease of the rubber tree in tropical regions of the world [28]. It is well-established that the expression level of the *cas5* gene in *Corynespora* varies across different growth stages of the pathogen in Hainan Province, China. Unlike *E. coli*, which regulates gene expression through multiple transcription factors to adapt to diverse soil environments [29,30,31,32], the regulatory mechanisms governing *cas5* gene expression during pathogenesis of *C. gloeosporioides* remains unclear. Gene expression in eukaryotes is regulated by a variety of conserved elements, and enzymes during transcription. Analysis of the *cas5* promoter revealed multiple TATA-boxes and CAAT-boxes. The TATA-box is a key site for the assembly of the transcription initiation complex, and the CAAT-box serves as a binding site for specific transcription factors [33,34,35,36]. Additionally, the two CpG islands of the promoter are potentially crucial for modulating gene expression through binding transcription factors and responding to DNA methylation status [37,38]. Studies have shown that CpG island binding protein BEND3 is highly enriched in the promoter regions of divalent genes and prevents premature activation of these genes during cell differentiation by binding to CpG islands [39].

Y1H can identify new gene functions or gene interactions through forward genetics approaches. The Y1H point-to-point verification test can determine the transcription factors that interact with the *cas5* gene, thereby providing insights into their function [40,41,42], but there are still some false positive events. 3-AT is a competitive inhibitor of histidine synthase in yeast. The addition of no more than 50 mM/L 3-AT in the medium can inhibit the background expression of His. At the same time, it can effectively reduce the self-activation phenomenon by truncating the promoter [43,44]. In this study, 30 candidate potential transcriptional regulators, classified into nine categories, were screened by the Y1H library. Previous studies have pointed out that transcription factors can regulate gene expression by modulating RNA polymerase II activity or exhibiting tissue-specific and stress-responsive expression patterns [45]. To clarify the function of transcription factors, CcbZIP3629, identified by Y1H point-to-point verification, was further analyzed. bZIP transcription factors are widely present in eukaryotes [46,47]. For example, in *Fusarium oxysporum* f.sp. *cubense* TR4, researchers identified 17 bZIP genes, which play a key role in the growth, stress response, and pathogenicity of the strain [48]. The *P. expansum* bZIP transcription factor PeAtf1, which causes postharvest blue mold of apple, can bind to the CRE motif and coordinate the expression of downstream genes related to virulence, secondary metabolism, and stress response by sensing environmental signals. The bZIP factor, which regulates the synthesis of toxins such as gibberellin by *Fusarium fujikuroi*, is essential for the complete pathogenicity of the pathogen. After knockout, the pathogenicity of the pathogen on rice was significantly reduced [49,50]. bZIP transcription factors regulate gene expression by binding to the ACGT core sequence in the promoter of the target gene and are involved in oxidative stress, nutrient utilization, and infection processes [51,52]. By combining the results of molecular docking, it is speculated that Cc-bZIP-3629 may also modulate transcriptional activity through interaction with the core sequence ACGT in the promoter. Combined with the results of promoter analysis, we speculate that CcbZIP3629 may also bind to the specific G-box/ABRE element on the *cas5* promoter to positively or negatively regulate the expression of *cas5* in response to signals such as host environment or oxidative stress, thereby fine-tuning the toxin synthesis during its infection. Bioinformatics analysis further suggests that CcbZIP3629 is a secreted protein and belongs to the bZIP transcription factor family. The protein contains multiple phosphorylation sites, typically located within functional domains. These sites can be phosphorylated by interaction with upstream kinases, thereby enhancing the stability of transcription factors and DNA binding ability [53]. Phosphorylation of OsbZIP72 at Ser71 by SAPK10 increases its binding efficiency to the promoter of the key jasmonic acid synthesis, promoting transcription in rice [54]. The typical characteristics of the bZIP family enable these transcription factors to interact with specific cis-regulatory motifs in the target gene promoter in the form of homologous or heterologous dimers, thereby activating or inhibiting transcription [55,56,57].

In the current study, a total of 30 potential transcription factors were screened by the Y1H library, and a bZIP transcription factor CcbZIP3629 was obtained by the Y1H point-to-point verification test. This transcriptional regulator may play an important role in the transcriptional regulation of the *cas5* gene. At present, we have only made a preliminary analysis of this transcriptional regulator, and its exact role in the regulation of *cas5* gene expression is still unclear. The molecular structure and function of this transcriptional regulator need to be further studied. These findings establish a framework for future investigations into the transcriptional regulation of the cas5 toxin.

## 5. Limitations and Future Prospects

This study provides a preliminary clue for the analysis of the transcriptional regulation mechanism of the key toxin gene *cas5* in *C*. *cassiicola*, but there are also some limitations that need to be clarified. First, yeast one-hybrid screening and molecular docking analysis are essentially in vitro or computational prediction methods. Although the results are enlightening, they are not completely equivalent to the real interaction in fungi. The former may have false positives, while the latter requires experiments to verify its binding mode and affinity. Secondly, the 1000 bp promoter fragment intercepted in the study was shorter, and the possible distal regulatory elements upstream or downstream were not included in the analysis, which limited the understanding of the complete regulatory pattern of the gene. Most importantly, there is no conclusive functional evidence that CcbZIP3629 directly binds to and regulates the *cas5* gene in pathogens.

In view of this, the core direction of future research should turn to in vivo verification and mechanism deepening. The primary task is to directly confirm the specific binding of CcbZIP3629 to the *cas5* promoter in cells by chromatin immunoprecipitation (ChIP-qPCR), and to clarify its regulatory function on toxin expression and pathogenicity at the physiological and pathological level by constructing gene knockout or overexpression strains. On this basis, we can further explore how the regulatory module responds to signals such as host environment or external stress (such as oxidative stress and nutrient fluctuations) to reveal its dynamic regulation. In addition, transcriptional regulation often involves multi-factor synergy. Subsequent work can explore other factors that interact or co-regulate with CcbZIP3629, thus moving from single-factor research to systematic analysis of regulatory networks, providing a more solid theoretical basis for new prevention and control strategies for this key virulence link.

## 6. Conclusions

To identify transcription factors regulating the *cas5* gene of the target spot pathogen of the rubber tree, we analyzed its promoter region and employed yeast one-hybrid screening. Using yeast one-hybrid (Y1H) technology, a bZIP family transcription factor CcbZIP3629 was screened, and point-to-point verification confirmed that it could directly bind to the promoter. Molecular docking predicted that CcbZIP3629 may regulate transcription by binding to specific sequences in the promoter. This finding provides a new target for further understanding the pathogenic molecular mechanism of the pathogen, and lays a theoretical foundation for the development of new disease prevention and control strategies based on interfering toxin regulation. The follow-up work will focus on the in vivo functional verification of this factor.

## Figures and Tables

**Table 1:** Primers used in this experiment.

Primer Name	Sequence (5′-3′)	Application
CDS III/3′ PCR Primer	ATGGCCATGGAGGCCAGTGAATTCGGG	Reverse transcription
SMART IV Oligonucleotide	CAGCTCGAGCTCGATGGATCCCTTTTTTTTTTTTTTTTTTTTTTTTTTTTTTVN	Reverse transcription
P1-F	ACGACGTACCAGATTACGCTCATATGGCCATGGAGGCCAGTGAATTCGGG	Ds cDNA amplification
P2-F	ACGACGTACCAGATTACGCTCATATGGCCATGGAGGCCAGTGAATTCAGGG	Ds cDNA amplification
P3-F	ACGACGTACCAGATTACGCTCATATGGCCATGGAGGCCAGTGAATTCAAGGG	Ds cDNA amplification
P4-R	CAGTATCTACGATTCATCTGCAGCTCGAGCTCGATGGATCCC	Ds cDNA amplification
Prime M1	AAGCAGTGGTATCAACGCAGAGT	Normalized
T7-F	TAATACGACTCACTATAGGGCGAG	PCR
AD-R	GCACGATGCACAGTTGAAG	PCR

## Data Availability

The genome sequence of the pathogen in this study has been deposited in the National Genomics Data Center (NGDC) under accession number GWHHJDM00000000.1, which is associated with BioSample number SAMC6273003. All other data generated in this study are available from the corresponding author upon request.

## References

[1] Feng R., Wang H., Zhang X., Li T., Huang C., Zhang S., Sun M., Shi C., Hu J., Gou J. (2024). Characteristics of *Corynespora cassiicola*, the causal agent of tobacco *Corynespora* leaf spot, revealed by genomic and metabolic phenomic analysis. Sci. Rep..

[2] Zeng T., Li C., Zhang B., Wang R., Fu W., Wang J., Zhang X. (2022). Rubber leaf disease recognition based on improved deep convolutional neural networks with a cross-scale attention mechanism. Front. Plant Sci..

[3] Lopez D., Ribeiro S., Label P., Fumanal B., Venisse J.-S., Kohler A., de Oliveira R.R., Labutti K., Lipzen A., Lail K. (2018). Genome-wide analysis of *Corynespora cassiicola* leaf fall disease putative effectors. Front. Microbiol..

[4] Déon M., Scomparin A., Tixier A., Mattos C.R.R., Leroy T., Seguin M., Roeckel-Drevet P., Pujade-Renaud V. (2012). First characterization of endophytic *Corynespora cassiicola* isolates with variant cassiicolin genes recovered from rubber trees in Brazil. Fungal Divers..

[5] Chau N.N.B., Van Minh N., Nghiep N.M., Vinh N.P., Nghia N.A., Quoc N.B. (2022). Identification and virulence evaluation of *Corynespora cassiicola* cassiicolin-encoding gene isolates from rubber trees in Vietnam. Trop. Plant Pathol..

[6] Jinji P., Xin Z., Yangxian Q., Yixian X., Huiqiang Z., He Z. (2007). First record of *Corynespora* leaf fall disease of *Hevea* rubber tree in China. Australas. Plant Dis. Notes.

[7] Reshma T.R., Babu S., Vineeth V.K., Philip S. (2024). Diversity of cassiicolin profiles and culture filtrate toxicity of *Corynespora cassiicola* isolates from south Indian rubber plantations. Ind. Crops Prod..

[8] Liu M., Ni Y., Zhao H., Liu X., Jia M., Liu H., Tian B. (2022). Molecular characterization of a novel victorivirus infecting *Corynespora cassiicola*. Arch. Virol..

[9] Silva W.P.K., Karunanayake E.H., Wijesundera R.L.C., Priyanka U.M.S. (2003). Genetic variation in *Corynespora cassiicola*: A possible relationship between host origin and virulence. Mycol. Res..

[10] Yang X., Guo Z., Yang Y., Abulaizi A., Xiong Z., Zhang S., Li B., Huang G. (2022). Isolation and identification of secondary metabolites produced by phytopathogenic fungus *Corynespora cassiicola* from *Hevea brasiliensis*. Molecules.

[11] Gao S., Zeng R., Xu L., Song Z., Gao P., Dai F. (2020). Genome sequence and spore germination-associated transcriptome analysis of *Corynespora cassiicola* from cucumber. BMC Microbiol..

[12] Déon M., Fumanal B., Gimenez S., Bieysse D., Oliveira R.R., Shuib S.S., Breton F., Elumalai S., Vida J.B., Seguin M. (2014). Diversity of the cassiicolin gene in *Corynespora cassiicola* and relation with the pathogenicity in *Hevea brasiliensis*. Fungal Biol..

[13] Ribeiro S., Tran D.M., Déon M., Clément-Demange A., Garcia D., Soumahoro M., Masson A., Pujade-Renaud V. (2019). Gene deletion of *Corynespora cassiicola* cassiicolin *cas1* suppresses virulence in the rubber tree. Fungal Genet. Biol..

[14] Li B., Yang Y., Cai J., Liu X., Shi T., Li C., Chen Y., Xu P., Huang G. (2021). Genomic characteristics and comparative genomics analysis of two Chinese *Corynespora cassiicola* strains causing *Corynespora* leaf fall (CLF) disease. J. Fungi.

[15] Knittel V., Vollmer I., Volk M., Dersch P. (2018). Discovering RNA-based regulatory systems for *Yersinia* virulence. Front. Cell. Infect. Microbiol..

[16] Li J., Liu W., Tian F., Tu Q., Xia X., Liu C., Zhang S., Ren H., Tong Y. (2021). First report of norovirus sequences isolated from raccoon dogs in mainland China. Virus Res..

[17] Song T., Ma Z., Shen D., Li Q., Li W., Su L., Ye T., Zhang M., Wang Y., Dou D. (2015). An oomycete CRN effector reprograms expression of plant *HSP* genes by targeting their promoters. PLoS Pathog..

[18] Yang H., Huang W., Fan S., Xue W., Liu Y., He Q., Song M., Wu W., Wang L.F., Lin C. (2025). Systematic characterization of the *bZIP* gene family in *Colletotrichum siamense* and functional analysis of three family members. Int. J. Biol. Macromol..

[19] Zhao Q., Shi Y., Wang Y., Xie X., Li L., Fan T., Guo L., Chai A., Li B. (2022). Temperature and humidity regulate sporulation of *Corynespora cassiicola* that is associated with pathogenicity in cucumber (*Cucumis sativus* L.). Biology.

[20] Ribeiro S., Label P., Garcia D., Montoro P., Pujade-Renaud V. (2021). Transcriptome profiling in susceptible and tolerant rubber tree clones in response to cassiicolin *cas1*, a necrotrophic effector from *Corynespora cassiicola*. PLoS ONE.

[21] Jiu S., Manzoor M.A., Chen B., Xu Y., Abdullah M., Zhang X., Lv Z., Zhu J., Cao J., Liu X.J. (2024). Chromosome-level genome assembly provides insights into the genetic diversity, evolution, and flower development of *Prunus conradinae*. Mol. Hortic..

[22] Lescot M., Déhais P., Thijs G., Marchal K., Moreau Y., Van de Peer Y., Rouzé P., Rombauts S. (2002). PlantCARE, a database of plant cis-acting regulatory elements and a portal to tools for in silico analysis of promoter sequences. Nucleic Acids Res..

[23] Xue J., Zhang H., Zhao Q., Cui S., Yu K., Sun R., Yu Y. (2023). Construction of yeast one-hybrid library of *Alternaria oxytropis* and screening of transcription factors regulating *swnk* gene expression. J. Fungi.

[24] Li L.C., Dahiya R. (2002). MethPrimer: Designing primers for methylation PCRs. Bioinformatics.

[25] Xu Y., Zhou J., Liu Q., Li K., Zhou Y. (2020). Construction and characterization of a high-quality cDNA library of *Cymbidium faberi* suitable for yeast one- and two-hybrid assays. BMC Biotechnol..

[26] Ma Y., Zhao X., Jia Y., Han Z., Yu C., Fan Z., Zhang Z., Xiao J., Zhao W., Bao Y. (2025). The updated genome warehouse: Enhancing data value, security, and usability to address data expansion. Genom. Proteom. Bioinform..

[27] CNCB-NGDC Members and Partners (2025). Database resources of the national genomics data center, China national center for bioinformation in 2025. Nucleic Acids Res..

[28] Seekham N., Kaewsalong N., Jantasorn A., Dethoup T. (2024). Biological control of *Corynespora* leaf fall disease in rubber by endophytic *Trichoderma* spp. under field conditions. Eur. J. Plant Pathol..

[29] Barthe P., Pujade-Renaud V., Breton F., Gargani D., Thai R., Roumestand C., de Lamotte F. (2007). Structural analysis of cassiicolin, a host-selective protein toxin from *Corynespora cassiicola*. J. Mol. Biol..

[30] Tran D.M., Clément-Demange A., Déon M., Garcia D., Le Guen V., Clément-Vidal A., Soumahoro M., Masson A., Label P., Le M.T. (2016). Genetic determinism of sensitivity to *Corynespora cassiicola* exudates in rubber tree (*Hevea brasiliensis*). PLoS ONE.

[31] Varela-Nájera R.G., De la Cruz M.A., Soria-Bustos J., González-Horta C., Delgado-Gardea M.C.E., Yáñez-Santos J.A., Cedillo M.L., Hirakawa H., Fox J.G., Sánchez-Ramírez B. (2025). The response regulator OMPR negatively controls the expression of genes implicated in tilimycin and tilivalline cytotoxin production in *Klebsiella oxytoca*. Microorganisms.

[32] Nakamoto S., Kobayashi I., Watanabe K., Kikuta T., Imamura S., Shimada T. (2025). Identification of a comprehensive set of transcriptional regulators involved in the long-term survivability of *Escherichia coli* in soil. Sci. Rep..

[33] Komissarov E.N., Diabankana R.G., Abdeeva I., Afordoanyi D.M., Gudkov S.V., Dvorianinova E.M., Bruskin S.A., Dmitriev A.A., Validov S.Z. (2025). Genomic differences between two *Fusarium oxysporum* formae speciales causing root rot in cucumber. J. Fungi.

[34] Nuss A.M., Heroven A.K., Dersch P. (2017). RNA regulators: Formidable modulators of *Yersinia* virulence. Trends in Microbiol..

[35] Yang Q.E., Ma X., Li M., Zhao M., Zeng L., He M., Deng H., Liao H., Rensing C., Friman V.-P. (2024). Evolution of triclosan resistance modulates bacterial permissiveness to multidrug resistance plasmids and phages. Nat. Commun..

[36] Blumenstein J., Dostálová H., Rucká L., Štěpánek V., Busche T., Kalinowski J., Pátek M., Barvík I. (2024). Promoter recognition specificity of *Corynebacterium glutamicum* stress response sigma factors σ^D^ and σ^H^ deciphered using computer modeling and point mutagenesis. J. Comput. Aided Mol. Des..

[37] Hughes J.R., Cheng J.-F., Ventress N., Prabhakar S., Clark K., Anguita E., De Gobbi M., de Jong P., Rubin E., Higgs D.R. (2005). Annotation of cis-regulatory elements by identification, subclassification, and functional assessment of multispecies conserved sequences. Proc. Natl. Acad. Sci. USA.

[38] Huang Y., Liu H., Du H., Zhang W., Kang X., Luo Y., Zhou X., Li L. (2019). Developmental features of DNA methylation in CPG islands of human gametes and preimplantation embryos. Exp. Ther. Med..

[39] Zhang J., Zhang Y., You Q., Huang C., Zhang T., Wang M., Zhang T., Yang X., Xiong J., Li Y. (2022). Highly enriched *bend3* prevents the premature activation of bivalent genes during differentiation. Science.

[40] Leighton J.C., Waterfall J.J., Gilchrist D.A., Fargo D.C., Kwak H., Adelman K., Lis J.T. (2012). Defining the status of RNA polymerase at promoters. Cell Rep..

[41] Sandelin A., Carninci P., Lenhard B., Ponjavic J., Hayashizaki Y., Hume D.A. (2007). Mammalian RNA polymerase II core promoters: Insights from genome-wide studies. Nat. Rev. Genet..

[42] Elango N., Yi S.V. (2011). Functional relevance of CPG island length for regulation of gene expression. Genetics.

[43] Fields S., Sternglanz R. (1994). The two-hybrid system: An assay for protein-protein interactions. Trends Genet..

[44] Edwards M.J., Thomas R.C. (2000). Protein phosphatase type 1-dependent dephosphorylation of the retinoblastoma tumor suppressor protein in ultraviolet-irradiated human skin and keratinocytes. J. Investig. Dermatol..

[45] Linzer N., Trumbull A., Nar R., Gibbons M.D., Yu D.T., Strouboulis J., Bungert J. (2021). Regulation of RNA polymerase ii transcription initiation and elongation by transcription factor TFII-I. Front. Mol. Biosci..

[46] Johnson D.S., Mortazavi A., Myers R.M., Wold B. (2007). Genome-wide mapping of in vivo protein-DNA interactions. Science.

[47] Cao Y., Bi M., Yang P., Song M., He G., Wang J., Yang Y., Xu L., Ming J. (2021). Construction of yeast one-hybrid library and screening of transcription factors regulating *LhMYBSPLATTER* expression in Asiatic hybrid lilies (*Lilium* spp.). BMC Plant Biol..

[48] Xie Y., Huang H., Huo Y., Yang W., Li Y., Liu S., Li C. (2025). Genome-wide profiling of *bZIP* transcription factors and focbzip11′s impact on Fusarium tr4 pathogenicity. Int. J. Mol. Sci..

[49] Wang Y., Wang K., Yang Q., Wang Z., Su Y., Chen X., Zhang H. (2025). Chromatin accessibility profile and the role of peatf1 transcription factor in the postharvest pathogen *Penicillium expansum*. Hortic. Res..

[50] Zhao K., Liu L., Huang S. (2022). Genome-wide identification and functional analysis of the *bZIP* transcription factor family in rice bakanae disease pathogen, *Fusarium fujikuroi*. Int. J. Mol. Sci..

[51] Liu L., Zhang Y., Wang Q., Tao X., Fang J., Zheng W., Zhu L., Jia B., Heng W., Li S. (2022). Identification of *bZIP* transcription factors and their responses to brown spot in pear. Genet. Mol. Biol..

[52] Han H., Wang C., Yang X., Wang L., Ye J., Xu F., Liao Y., Zhang W. (2023). Role of *bZIP* transcription factors in the regulation of plant secondary metabolism. Planta.

[53] Yamaoka Y., Shin S., Choi B.Y., Kim H., Jang S., Kajikawa M., Yamano T., Kong F., Légeret B., Fukuzawa H. (2019). The *bZIP1* transcription factor regulates lipid remodeling and contributes to er stress management in *Chlamydomonas reinhardtii*. Plant Cell.

[54] Wang Y., Hou Y., Qiu J., Wang H., Wang S., Tang L., Tong X., Zhang J. (2020). Abscisic acid promotes jasmonic acid biosynthesis via a ‘SAPK10-bZIP72-AOC’ pathway to synergistically inhibit seed germination in rice (*Oryza sativa*). New Phytol..

[55] Duan L., Mo Z., Fan Y., Li K., Yang M., Li D., Ke Y., Zhang Q., Wang F., Fan Y. (2022). Genome-wide identification and expression analysis of the *bZIP* transcription factor family genes in response to abiotic stress in *Nicotiana tabacum* L.. BMC Genom..

[56] Wu C., Shan W., Liu X., Zhu L., Wei W., Yang Y., Guo Y., Bouzayen M., Chen J., Lu W. (2022). Phosphorylation of transcription factor *bZIP21* by map kinase MPK6-3 enhances banana fruit ripening. Plant Physiol..

[57] Sirichandra C., Davanture M., Turk B.E., Zivy M., Valot B., Leung J., Merlot S. (2010). The arabidopsis ABA-activated kinase OST1 phosphorylates the *bZIP* transcription factor ABF3 and creates a 14-3-3 binding site involved in its turnover. PLoS ONE.

